# Association of Dietary Approaches to Stop Hypertension diet and Mediterranean diet with blood pressure in less-developed ethnic minority regions

**DOI:** 10.1017/S1368980022000106

**Published:** 2022-12

**Authors:** Suyao Dai, Xiong Xiao, Chuanzhi Xu, Yan Jiao, Zixiu Qin, Jiantong Meng, Haojiang Zuo, Peibin Zeng, Dan Tang, Xinyu Wu, Qucuo Nima, Deji Quzong, Xing Zhao

**Affiliations:** 1 West China School of Public Health and West China Fourth Hospital, Sichuan University, No. 16, Section 3, Renmin South Road, Chengdu, Sichuan 610041, China; 2 School of Public Health, Kunming Medical University, Kunming, Yunnan, China; 3 Chongqing Municipal Centre for Disease Control and Prevention, Chongqing, China; 4 School of Public Health, the Key Laboratory of Environmental Pollution Monitoring and Disease Control, Ministry of Education, Guizhou Medical University, Guiyang, Guizhou, China; 5 Chengdu Center for Disease Control and Prevention, Chengdu, Sichuan, China; 6 Tibet Centre for Disease Control and Prevention CN, No. 21, Linkuo North Road, Chengguan District, Lhasa, Tibet Autonomous Region 850000, China; 7 Tibet University, No.10, East Tibet University Road, Lhasa, Tibet Autonomous Region 850000, China

**Keywords:** DASH-style diet, Mediterranean-style diet, Blood pressure, Epidemiology, Hypertension

## Abstract

**Objective:**

We aimed to investigate the associations of Dietary Approaches to Stop Hypertension (DASH)-style diet and Mediterranean-style diet with blood pressure (BP) in less-developed ethnic minority regions (LEMR).

**Design:**

Cross-sectional study.

**Setting:**

Dietary intakes were assessed by a validated FFQ. Dietary quality was assessed by the DASH-style diet score and the alternative Mediterranean-style diet (aMED) score. The association between dietary quality and BP was evaluated using multivariate linear regression model. We further examined those associations in subgroups of BP level.

**Participants:**

A total of 81 433 adults from the China Multi-Ethnic Cohort (CMEC) study were included in this study.

**Results:**

In the overall population, compared with the lowest quintile, the highest quintile of DASH-style diet score was negatively associated with systolic BP (SBP) (coefficient –2·78, 95 % CI –3·15, –2·41; *P*_for trend_ < 0·001), while the highest quintile of aMED score had a weaker negative association with SBP (coefficient –1·43, 95 % CI –1·81, –1·05; *P*_for trend_ < 0·001). Both dietary indices also showed a weaker effect on diastolic BP (coefficient for DASH-style diet –1·06, 95 % CI –1·30, –0·82; coefficient for aMED –0·43, 95 % CI –0·68, –0·19). In the subgroup analysis, both dietary indices showed a stronger beneficial effect on SBP in the hypertension group than in either of the other subgroups.

**Conclusion:**

Our results indicated that the healthy diet originating from Western developed countries can also have beneficial effects on BP in LEMR. DASH-style diet may be a more appropriate recommendation than aMED as part of a dietary strategy to control BP, especially in hypertensive patients.

## Graphical abstract

**Figure:**
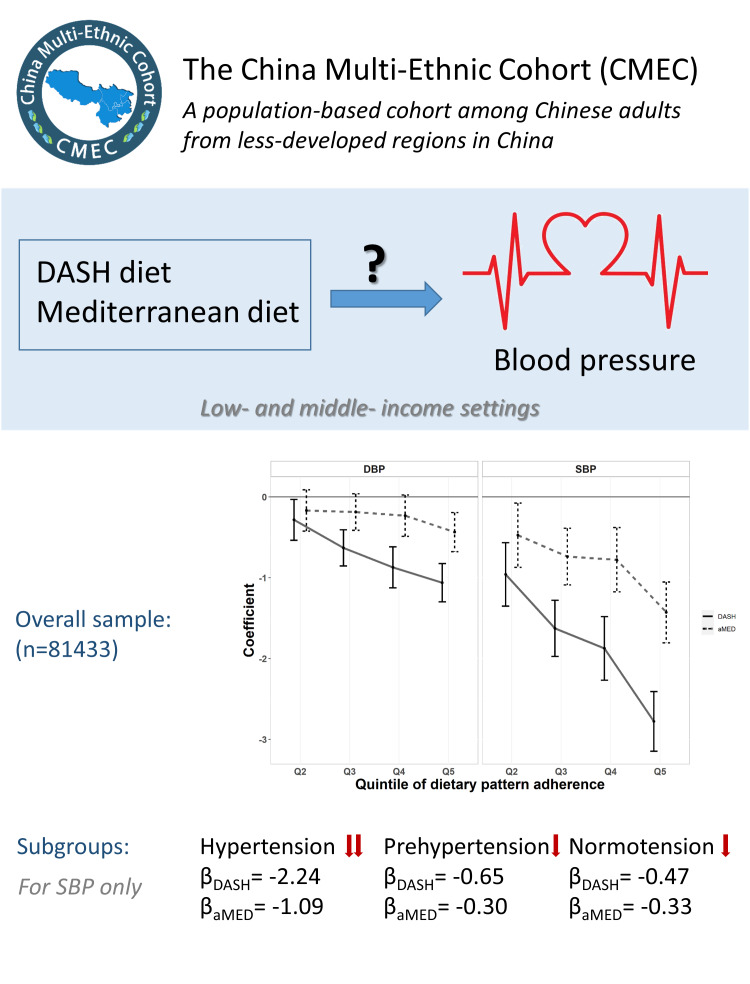

High blood pressure (BP) is the top risk factor for many non-communicable diseases (NCD), and almost one-fifth of global deaths in 2019 were attributed to high BP^(1)^. There is no evidence for a sharp risk threshold, instead, CVD mortality increases progressively over the range of BP, from normotension to prehypertension and finally hypertension^(2)^. Thus, NCD Countdown 2030 highlighted policies and interventions to reduce BP in pursuit of a substantial (one-third) reduction in the prevalence of high BP relative to 2015^(3)^.

The number of adults with elevated BP has increased more rapidly in low- and middle-income countries than in high-income countries over the past decades, as well as in populations of low socio-economic status and racial/ethnic minorities^(4–7)^. Although antihypertensive medications seem to be the most important approach to control BP and reduce cardiovascular events, the unavailability and unaffordability of BP-lowering medicine makes it arduous to optimally control BP in less-developed regions^(8)^. Given the prominent role of diet in BP homeostasis^(9)^, dietary strategies are feasible and acceptable measures to control BP and are recommended by the American College of Cardiology/American Heart Association in High Blood Pressure Clinical Practice Guidelines; one such strategy is the Dietary Approaches to Stop Hypertension (DASH) diet^(10)^. The Mediterranean diet (MED) has also been demonstrated to benefit BP in populations from Western developed countries^(11)^. Previous studies exploring the association between dietary quality (DASH and MED) and BP were mainly conducted in developed countries^(12,13)^. Valid evidence for the BP-lowering effect of a medically beneficial diet is scarce in racial/ethnic minority groups in less-developed regions, whose dietary habits differ substantially^(14,15)^. Furthermore, dietary factors could play diverse roles in different BP levels to better inform dietary intervention strategies. Dietary changes could not only lower BP and potentially prevent hypertension in non-hypertensive individuals but also serve as initial treatment before the start of drug therapy in stage I hypertension, further lowering BP and facilitating medication step-down among hypertensive individuals already on drug therapy^(9)^. Understanding the association between dietary quality and BP in different levels of BP can help facilitate the integration of guideline to recommend certain dietary modifications in three levels of prevention of hypertension. However, few studies have investigated the efficacy of diet on BP in subgroups of BP in less-developed regions.

To address these gaps in knowledge, this study conducted a cross-sectional analysis based on the China Multi-Ethnic Cohort (CMEC), which is a large community population-based cohort undertaken in less developed Southwestern China^(16)^. This cohort has a heavy burden of CVD and great diversity in socio-economic status, habitual diet, cultural background, living environment, etc. Therefore, the CMEC study provides a unique opportunity to explore the association between diet and BP in less-developed ethnic minority regions (LEMR). We aimed to (1) examine the association between adherence to DASH-style diet and Mediterranean-style diet with BP and (2) further explore the association between dietary quality and BP in subgroups (normotension, prehypertension, and hypertension).

## Materials and methods

### Study population

This study is a subsample of the CMEC study, which is an ongoing cohort study of 99 556 participants aged 18–79 years. The details of the study design, sampling strategy, and baseline characteristics are described elsewhere^(16)^. The baseline survey included a tablet-based electronic questionnaire administered in a face-to-face interview, anthropometric measurements, a thorough medical examination, and blood and urine tests. All the measurements were conducted according to standard operating procedures by qualified health workers.

In the present study, we excluded participants under 30 or over 79 years of age (*n* 1003), those with missing information on diet-related variables (*n* 343), those with missing information on outcome-related data (*n* 4302), those with implausible BMI values (BMI < 14 or > 45 kg/m^2^, *n* 172) and those with unusual daily energy intakes (< 600 or > 3500 kcal/d for females, < 800 or > 4200 kcal/d for males) (*n* 2284). We also excluded 10 019 participants with self-reported physician-diagnosed hypertension and use of antihypertensive medication at the baseline survey. Participants who reported being hypertensive but not taking any antihypertensive drugs were included. The final sample consisted of 81 433 participants. A flowchart of the selection process for the study population is shown in Supplemental Fig. 1s.

### Assessment of dietary intakes

At the baseline survey, a validated quantitative FFQ was used to assess dietary intake; the questionnaire was administered by trained staff using standard container (standard bowl or cup, different types of foods have been pre-measured by the standard containers to give reference levels). The FFQ included thirteen major food groups according to the Chinese Dietary Guidelines and the eating habits of Southwestern Chinese. For each food group, the participants were asked to report the quantity (average number of grams per meal) and frequency (four frequency categories in units ranging from times per day to times per year) of consumption during the past 12 months. Daily consumption of each food group was calculated by multiplying the daily consumption frequency by the average quantity per meal. In the case of dairy products, a person reported that he or she drunk milk two times per d and drank about 250 g every time; thus, his or her daily intake of dairy products is equal to two times multiplying by 250 g each time, which is equivalent to 500 g per d. The salt intake was used as a proxy for the Na intake, and the individual-level intake of salt was calculated according to the salt consumed at the household level per month. The total daily energy intake was estimated according to the China food exchange lists and the 2018 China food composition tables (standard edition)^(17,18)^. Detailed information about our dietary assessment tool has been reported previously^(19)^.

### Assessment of dietary quality

Given that the DASH diet recommendations and original Mediterranean-style diet were developed based on the Western style diet; those recommendations cannot be directly used to assess the dietary quality in LEMR. In this study, we used the DASH-style diet and alternative Mediterranean-style diet to assess the dietary quality based on the local food patterns in LEMR.

Considering that the consumption of non-fat and low-fat dairy was extremely low in our study population, adherence to a DASH-style diet was assessed with a modified DASH score^(20,21)^ in which non-fat and low-fat dairy products were replaced with full-fat dairy products. In addition, as the regular consumption of sweetened beverages in our study population was only 7·2 %, we excluded the food group component of sweetened beverages. Thus, the DASH-style diet is characterised by a high intake of fruits, vegetables, legumes, dairy products, and whole grains and a low intake of Na and red and processed meats. For each of the presumed healthy food group components of DASH, the maximum score (5) was assigned to those in the highest quintile, and the minimum score (1) was assigned to those in the lowest quintile, with intermediate values being assigned accordingly. However, for the presumed unhealthy foods, values were assigned in the opposite manner. Thus, the total DASH score ranged from 7 (minimal adherence to the DASH-style diet) to 35 (maximal adherence).

An alternative Mediterranean-style diet (aMED) score that reflects an adapted version of the traditional MED for the non-Greek population^(22–24)^ was used to evaluate the degree of adherence to the Mediterranean-style diet. We eliminated the food group component of nuts because we did not collect information on nut consumption. Thus, this score included eight nutritional components of the Mediterranean-style diet, characterised by: a high intake of vegetables, legumes, fruits, cereals, fish and foods with a high ratio of unsaturated to saturated fats; a low intake of poultry, meat and meat products; and a regular but moderate intake of alcohol. For each of the eight food group components of aMED except alcohol consumption, we categorised the food group consumption into sex-specific quintiles and scored all participants from 1 to 5 according to their intake ranking. For alcohol consumption (g/d), we used sex-specific presumed cut-offs^(23)^. Thus, the total aMED score ranged from 8 (minimal adherence to the aMED) to 40 (maximal adherence).The detailed scoring criteria for the DASH-style diet and aMED are described in the supplemental material (see online supplementary material, Supplemental Table 1s & 2s).

### Assessment of outcomes

Systolic BP (SBP) and diastolic BP (DBP) were measured three times with an Omron HEM-8711 BP monitor after 5 min of rest in a seated position; these measurements were conducted by trained staff members according to standardised protocols. If an unusual BP measurement was detected, a mercury sphygmomanometer was used to verify the unusual measurement. The mean of the three measurements was calculated for use in the analysis.

The levels of BP in this study were divided into three subgroups: normotension, prehypertension and hypertension. In accordance with the Seventh Report of the Joint National Committee on Prevention, Detection, Evaluation, and Treatment of High Blood Pressure (the JNC 7 report)^(25)^, hypertension was defined as average SBP ≥ 140 mm Hg or DBP ≥ 90 mm Hg. Prehypertension was defined as 120 mm Hg ≤ SBP < 140 mm Hg or 80 mm Hg ≤ SBP < 90 mm Hg. Normotension was defined as 90 mm Hg ≤ SBP < 120 mm Hg and 60 mm Hg ≤ DBP < 80 mm Hg. Hypotension was defined as SBP < 90 mm Hg or DBP < 60 mm Hg. Considering that the hypotensive population was relatively small in our sample (*n* 281) and that the distribution of BP in the hypotensive group was negatively skewed, the hypotension group was merged with the normotension group in the final analysis.

### Assessment of covariates

We obtained information on covariates from the baseline questionnaire, which included nine sections, that is, demographics and socio-economic status, smoking and indoor air pollution, alcohol consumption, tea and other beverage consumption, health status (quality of life, self-rated health status, personal and family disease history), physical activity, reproductive history (only for women), and mental health status (life events, sleep, psychological conditions and social support). To identify the potential confounders, we constructed a directed acyclic graph (DAG) under the protocol of ‘Evidence Synthesis for Constructing Directed Acyclic Graphs’ (ESC-DAGs), which combines evidence synthesis strategies and causal inference principles^(26)^. We then ran independent tests to continuously modify the proposed DAG until the implied conditional independences were achieved (see online supplementary material, Supplemental Text 1s). According to the causal diagram and back door criteria^(27)^, we adjusted the models for sex (male or female), age (years), urbanicity (urban or rural), ethnicity (Han, Tibetan, Yi, Miao, Bai, Bouyei, Dong), marital status (married/cohabiting or not), highest education attained (no formal school, primary school, middle or high school, college/university or higher), household income (< ¥12 000, ¥12000–19999, ¥20000–5999, ¥60000–99999, ¥100000–199999, > ¥200 000), profession (agriculture, manufacturing, service, unemployed or other), regular smoking (never, former and current), physical activity in metabolic equivalent of task (MET) and hours per d, total energy intake (quintiles), regular intake of sweetened beverages (never, former and current), regular intake of dietary supplements (yes or no), regular intake of spicy food (yes or no), regular intake of pepper foods (yes or no), BMI (< 24, 24–28, ≥ 28), insomnia symptoms (presence or absence), depression symptoms (presence or absence), anxiety symptoms (presence or absence), menopause status for women (premenopausal, perimenopausal and postmenopausal), and family history of hypertension (yes or no).

### Statistical analysis

A descriptive analysis of the baseline characteristics of the overall study sample, as well as stratification by quintiles of DASH-style diet and aMED scores, was performed. Categorical variables are expressed as frequencies and proportions (%), and continuous variables are presented as the means and standard deviations with adjustment for age and sex by direct standardisation (using the entire study sample as the standard population). Continuous and categorical demographic variables across groups were compared using ANOVA and chi-square tests, respectively.

We used multivariate linear regression to estimate the separate associations between the dietary quality scores and BP (SBP and DBP) overall, as well as in normotensive, prehypertensive and hypertensive subgroups, after adjusting for the confounders identified by the DAG. Each of the dietary quality scores was categorised into quintiles for the entire study population, with the lowest fifth of dietary quality scores as the reference group. To test for linear trends, we assigned a median value to each quintile of diet scores, producing a single continuous variable used in the model. To evaluate the relative influence of each of the dietary components on BP in association with DASH-style diet and aMED, we subtracted one component at a time from the original score and estimated the BP coefficient of each diet score^(28)^. Owing to the built-in quality control in the tablet-based questionnaire and the stringent data audit, the proportion of missing data in this study is very low, and we set most of the missing values from unverifiable outliers to ‘NA’ after reviewing audio of the interviews. For missing values of food group intake, we performed multiple imputation (with five imputations) by chained equations^(29)^.

In addition, we conducted stratified analysis among pre-defined strata of variables including sex, age, BMI, urbanicity, regular smoking and physical activity to examine potential effect modifiers. Heterogeneity among different strata was assessed with the *I* ^2^ statistic and the chi-square test (*α* = 0·1, *I* ^2^ > 60 % was considered significantly heterogeneous). To test the robustness of our findings, we also performed several sensitivity analyses. First, we included all participants in our cohort who reported having hypertension and using antihypertensive medication to examine the magnitude of potential reverse causality. Second, we used a more stringent exclusion criterion by excluding patients who reported pre-existing physician-diagnosed hypertension, diabetes, hyperlipidemia, CHD, stroke or cancer before the main analyses. Third, we ran a complete case analysis without multiple imputation of the ‘NA’ values of dietary intake. Finally, we used a spline to examine the potential non-linearity of the relationship between BP and diet scores (treated as a continuous variable).

All analyses were performed with R version 4.0.2 (R Project for Statistical Computing, Vienna, Austria). We followed the reporting guidelines of the Strengthening the Reporting of Observational Studies in Epidemiology (STROBE) Statement for cross-sectional studies.

## Results

### Baseline characteristics

The final study sample included 81 433 participants. Overall, the mean age was 50·5 years (sd = 11·2), 60·1 % of participants were women, 64·1 % of participants lived in rural areas and 11·6 % of people had a university-level education or above. We ascertained 32 155 cases of normotension, 30 760 cases of prehypertension and 18 518 cases of hypertension. Table 1 showed the sex- and age-adjusted characteristics of the study participants at baseline in the overall sample and stratified by quintiles of DASH-style diet and aMED scores. Participants in the highest quintile of both diet scores were more likely to be younger and female and tended to have higher socio-economic status (education, urban residence and household income) and have lower total physical activity; they were less likely to report mental disorders but more likely to have a family history of hypertension.

**Table 1:** Age- and sex-standardised baseline characteristics in the CMEC study, according to quintiles of DASH-style diet score and aMED score

Characteristic			DASH					aMED				
	Overall *n* 81 433		Q1		Q5			Q1		Q5		
	*n*	%	*n*	%	*n*	%	*P* value	*n*	%	*n*	%	*P* value
Age (years)	81 433											
Mean	50·5		52·5		49·5		< 0·001	52·3		49·8		< 0·001
sd	11·2		11·1		11·6			11·7		10·9		
Female sex (%)	49 387	60·6	52·9		67·4		< 0·001	51·1		66·4		< 0·001
Urban residence (%)	29 268	35·9	26·5		50·0		< 0·001	22·8		50·9		< 0·001
Ethnic group (%)*							< 0·001					< 0·001
Sichuan Basin	38 857	47·7	36·4		63·6			28·9		66·0		
Yunnan–Guizhou Plateau	33 884	41·6	55·5		28·2			50·3		31·7		
Qinghai–Tibet Plateau	8692	10·7	8·1		8·2			20·8		2·3		
Married or cohabiting (%)	72 858	89·5	88·6		90·1		< 0·001	88·9		90·3		< 0·001
Highest education completed (%)							< 0·001					< 0·001
No formal school	20 752	25·5	37·2		14·7			41·7		13·3		
Primary school	20 432	25·1	28·1		20·5			27·6		21·8		
Middle and high school	30 797	37·8	28·4		46·8			24·2		49·2		
College or university	9451	11·6	6·3		18·0			6·5		15·7		
Household income (yuan/year) (%)							< 0·001					< 0·001
< 12 000	13 425	16·5	25·8		9·8			25·7		9·6		
12 000–19 999	14 771	18·2	21·1		14·5			23·5		13·7		
20 000–59 999	29 804	36·6	34·8		35·8			34·3		37·3		
60 000–99 999	12 096	14·9	10·5		19·7			9·2		19·6		
100 000–199 999	8974	11·0	6·3		15·5			5·9		15·4		
> 200 000	2263	2·8	1·3		4·6			1·4		4·3		
Occupation (%)†							< 0·001					< 0·001
Primary industry practitioner	27 728	34·1	44·6		23·2			48·5		23·0		
Secondary industry practitioner	6213	7·6	8·4		7·2			6·9		7·9		
Tertiary industry practitioner	31 010	38·1	32·4		42·3			29·8		42·8		
Unemployed	16 416	20·2	14·5		27·2			14·8		26·3		
Regular smoking (%)							< 0·001					< 0·001
Never	61 258	75·2	74·7		76·6			77·0		74·2		
Previous	16 375	20·1	21·8		17·7			19·2		19·9		
Current	3800	4·7	3·5		5·7			3·8		5·9		
Total physical activity (MET h/d)	81 092											
Mean	26·7		29·1		24·4		< 0·001	28·7		25·2		< 0·001
sd	18·3		19·7		16·7			20·0		16·7		
BMI (kg/m^2^)	81 433											
Mean	24·1		24·2		23·9		< 0·001	24·2		24·1		0·002
sd	3·4		3·6		3·3			3·7		3·3		
Dietary supplements (%)‡	12 723	15·6	9·1		22·7		< 0·001	8·6		21·5		< 0·001
Regular sweetened beverage intake (%)§							0·194					< 0·001
Never	75 174	92·3	93·3		93·5			87·4		95·5		
Previous	365	0·4	0·4		0·5			0·4		0·5		
Current	5894	7·2	6·3		6·0			12·2		4·1		
Regular spicy food intake (%)	48 708	59·8	65·9		54·2		< 0·001	57·4		60·6		< 0·001
Regular pepper intake (%)	33 767	41·5	34·6		44·5		< 0·001	31·8		47·7		< 0·001
Insomnia symptoms (%)	35 459	43·6	47·7		40·4		< 0·001	46·3		41·7		< 0·001
Depression symptom (%)	3897	4·8	7·5		3·4		< 0·001	6·4		3·6		< 0·001
Anxiety symptoms (%)	4576	5·6	9·2		3·5		< 0·001	7·9		3·8		< 0·001
Menopausal status in women (%)							0·070					0·150
Premenopausal	24 020	48·6	47·6		48·8			48·4		48·4		
Perimenopausal	3474	7·0	6·8		7·2			6·9		7·6		
Postmenopausal	21 893	44·3	45·6		44·1			44·7		44·0		
Family history of hypertension (%)	23 646	29·0	23·6		34·5		< 0·001	20·8		35·0		< 0·001
SBP	81 433											
Mean	124·1		125·8		123·0		< 0·001	125·0		123·8		< 0·001
sd	18·3		17·6		16·5			17·3		16·7		
DBP	81 433											
Mean	78·6		79·5		77·7		< 0·001	79·1		78·1		< 0·001
sd	11·0		11·1		10·5			11·0		10·6		
Blood pressure level (%)							< 0·001					< 0·001
Normotension	32 155	39·5	35·9		41·8			37·4		40·3		
Prehypertension	30 760	37·8	38·4		37·8			38·4		38·0		
Hypertension	18 518	22·7	25·7		20·4			24·2		21·7		

CMEC, China Multi-Ethnic Cohort; DASH, Dietary Approaches to Stop Hypertension; aMED, alternative Mediterranean-style diet; MET, metabolic equivalent of task; Q, quartile; SBP, systolic blood pressure; DBP, diastolic blood pressure.

* We aggregated various ethnic groups into three geographic regions due to their high similarity in diet and baseline characteristics.

† Primary industry practitioners are defined as workers in the farming, forestry, animal husbandry and fishery industries. Secondary industry practitioners are defined as workers in processing and manufacturing industries. Tertiary industry practitioners are defined as workers in industries other than primary and secondary industries.

‡ Dietary supplements refer to fish liver oil, vitamins and calcium tablets.

§ Previous regular sweetened beverage intake is defined as past consumption of sweetened beverages every week for more than half a year. Current regular sweetened beverage intake is defined as an average of at least 1–2 d of consumption per week in the past year.

### Association between diet scores and blood pressure

**Fig. 1:**
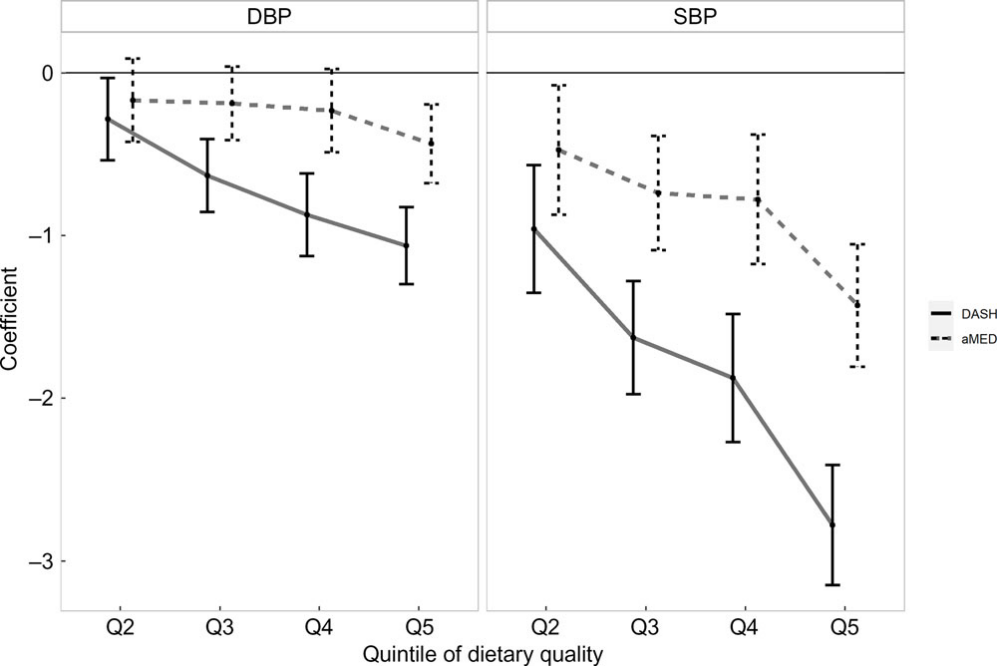
Association between DASH-style diet and aMED scores and blood pressure in overall samples. DASH, Dietary Approaches to Stop Hypertension; aMED, alternative Mediterranean-style diet; SBP, systolic blood pressure; DBP, diastolic blood pressure; Q, quintile

Associations of DASH-style diet and aMED scores with SBP and DBP in the overall samples are shown in Fig. 1. Although both dietary indices had inverse linear associations with BP, DASH-style diet showed a stronger association with both SBP and DBP than aMED did (all *P* values for trend < 0·001). In a comparison between the highest and lowest quintiles of diet scores after adjustment for multiple potential confounding factors, the DASH-style diet score was associated with a 2·78-mm Hg reduction in SBP (95 % CI –3·15, –2·41) and an 1·06-mm Hg reduction in DBP (95 % CI –1·30, –0·82). Similarly, the aMED score was associated with a 1·43-mm Hg reduction in SBP (95 % CI –1·81, –1·05) and a 0·44-mm Hg reduction in DBP (95 % CI –0·68, –0·19). Our results also indicated that SBP had a stronger negative association with dietary quality than DBP had.

Table 2 presents the associations of DASH-style diet and aMED scores with BP stratified by BP level. With the lowest quintile as a reference, the highest quintile of both DASH-style diet and aMED scores showed a stronger association with SBP in the hypertension subgroup than in either of the other BP subgroups (*β*_DASH_ = –2·24, 95 % CI –2·89, –1·58; *β*_aMED_ = –1·09, 95 % CI –1·75, –0·43). No such association was observed between either diet score or DBP. Our results also indicated that there was a stronger association between the highest quintile of DASH-style diet and SBP than that of aMED in all subgroups. However, some of these associations in other quantiles instead of Q5 were not statistically significant, it might be due to the weak association and lack of power. Although the DASH-style diet and aMED have some food group components in common, adherence to DASH-style diet consistently showed a larger effect on BP than adherence to aMED in the overall sample as well as in subgroups.

**Table 2:** Association of the DASH-style diet and aMED scores with blood pressure in subgroups of blood pressure level*

Coefficient	Quintiles of diet scores									*P*_for trend_ †
		Q2		Q3		Q4		Q5		
	Q1	*β*	95 % CI	*β*	95 % CI	*β*	95 % CI	*β*	95 % CI	
Normotension										
DASH										
SBP	Ref.	0·16	–0·14,0·46	−0·10	–0·36,0·16	−0·27	–0·56,0·02	−0·47	–0·74,–0·20	< 0·001
DBP	Ref.	0·24	–0·01,0·49	0·13	–0·09,0·35	−0·12	–0·37,0·12	0·08	–0·15,0·31	< 0·001
Participants, *n*	4975	4113		8052		5429		9459		
aMED										
SBP	Ref.	0·03	–0·27,0·32	−0·03	–0·29,0·24	0·01	–0·29,0·30	−0·33	–0·60,–0·05	0·147
DBP	Ref.	0·13	–0·12,0·38	0·13	–0·09,0·34	0·13	–0·11,0·37	0·09	–0·14,0·32	< 0·001
Participants, *n*	4981	4225		8345		5456		9021		
Prehypertension										
DASH										
SBP	Ref.	−0·40	–0·66,–0·14	−0·44	–0·67,–0·20	−0·55	–0·81,–0·29	−0·65	–0·89,–0·40	< 0·001
DBP	Ref.	0·22	–0·02,0·45	0·14	–0·07,0·35	0·24	–0·00,0·48	0·18	–0·04,0·40	< 0·001
Participants, *n*	5929	4369		7731		4924		7674		
aMED										
SBP	Ref.	−0·09	–0·36,0·17	−0·28	–0·52,–0·05	−0·14	–0·41,0·13	−0·30	–0·55,–0·05	< 0·001
DBP	Ref.	0·03	–0·21,0·27	0·11	–0·10,0·32	0·06	–0·18,0·31	0·05	–0·18,0·27	< 0·001
Participants, *n*	5661	4307		7847		4930		7882		
Hypertension										
DASH										
SBP	Ref.	−1·09	–1·74,–0·43	−1·36	–1·96,–0·77	−1·18	–1·87,–0·49	−2·24	–2·89,–1·58	< 0·001
DBP	Ref.	−0·38	–0·82,0·05	−0·15	–0·55,0·24	−0·49	–0·95,–0·03	−0·25	–0·68,0·19	< 0·001
Participants, *n*	4320	2870		4599		2703		3944		
aMED										
SBP	Ref.	−0·26	–0·94,0·43	−0·92	–1·52,–0·31	−1·14	–1·84,–0·44	−1·09	–1·75,–0·43	< 0·001
DBP	Ref.	−0·14	–0·60,0·31	−0·18	–0·58,0·23	−0·15	–0·61,0·32	0·08	–0·36,0·52	< 0·001
Participants, *n*	3901	2667		4659		2899		4310		

DASH, Dietary Approaches to Stop Hypertension; SBP, systolic blood pressure; DBP, diastolic blood pressure; aMED, alternative Mediterranean-style diet.

* Multivariate linear regression with blood pressure as a dependent variable and quintile of the dietary quality score as an independent variable was used. Adjusted for sex, age, urbanicity, ethnicity, marital status, highest education attained, household income, profession, regular smoking, physical activity, BMI, total energy intake, regular intake of sweetened beverages, regular intake of dietary supplements, regular intake of spicy food, regular intake of pepper foods, insomnia symptoms, depression symptoms, anxiety symptoms, menopause status for women and family history of hypertension.

† Tests for linear trend based on variable assigned median value for each quintile.

The discrepant results between DASH-style diet and aMED were confirmed by food group analyses (see online supplementary material, Supplemental Table 3s & 4s). Among the discrepant food group components, the dairy product component, included in DASH-style diet but not in aMED, accounted for a majority of the beneficial effects of DASH-style diet on BP (33·63 % for SBP and 33·97 % for DBP); in contrast, the MUFA:SFA ratio, included in aMED but not in DASH-style diet, showed harmful effects on BP (–24·00 % for SBP and –63·38 % for DBP).

### Stratified and sensitivity analyses

**Fig. 2:**
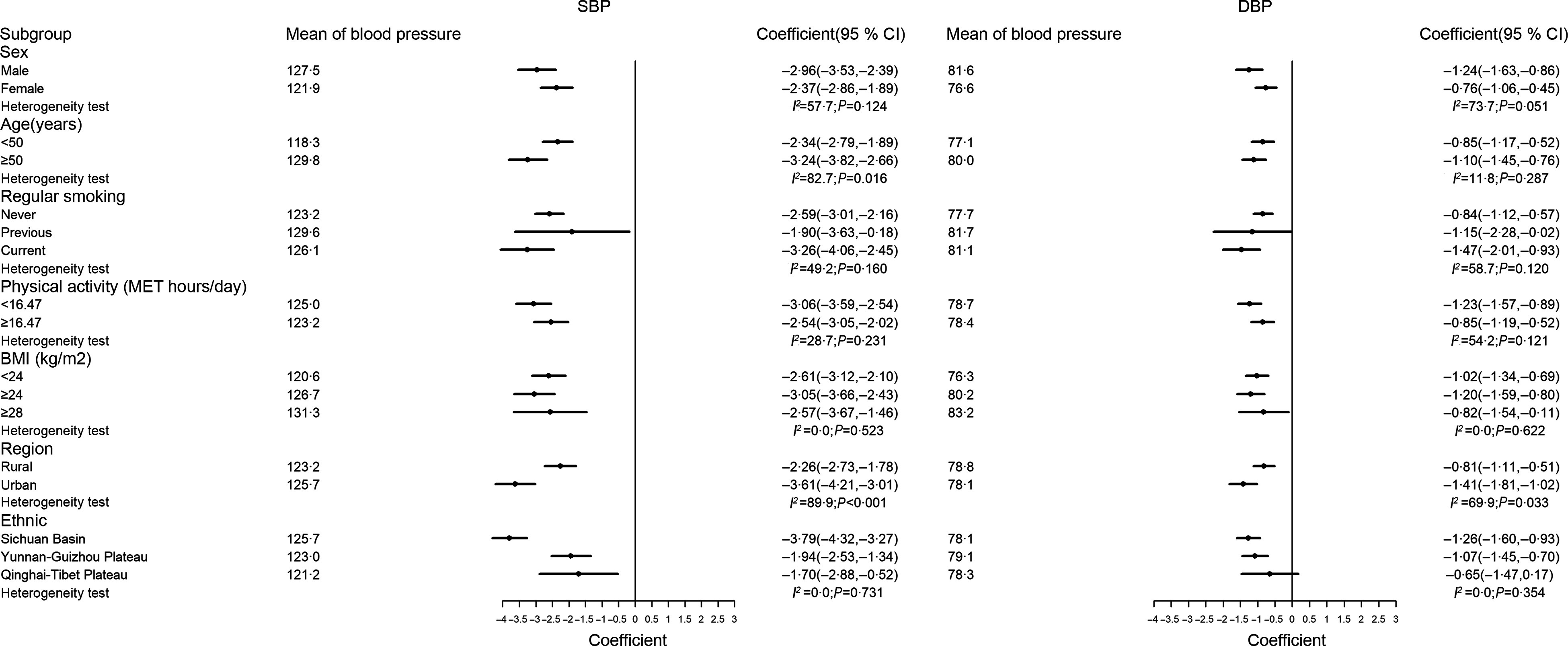
Association between DASH-style diet and blood pressure stratified by sex, age, smoking, physical activity, BMI, region and ethnic. DASH, Dietary Approaches to Stop Hypertension; SBP, systolic blood pressure; DBP, diastolic blood pressure

**Fig. 3:**
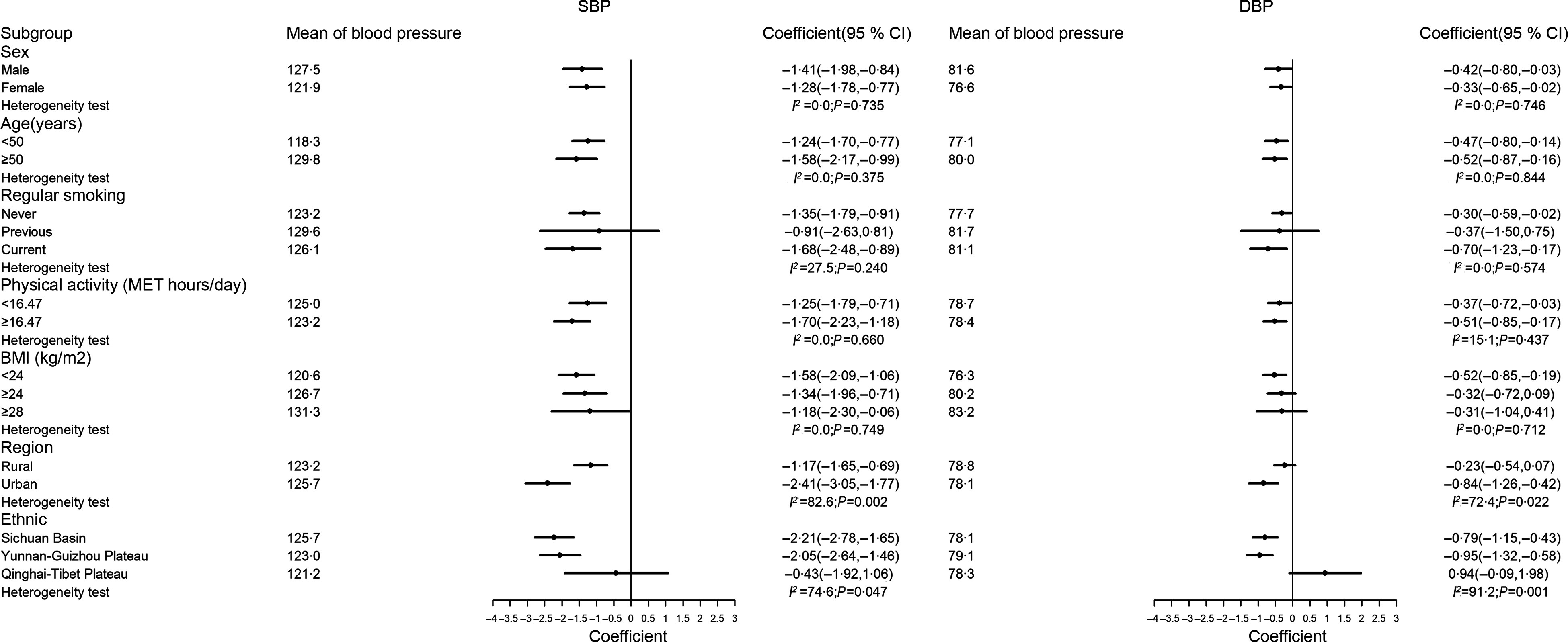
Association between aMED and blood pressure stratified by sex, age, smoking, physical activity, BMI, region and ethnic. aMED, alternative Mediterranean-style diet; SBP, systolic blood pressure; DBP, diastolic blood pressure

For simplicity, stratified analyses focused only on the association between dietary quality scores and BP in the overall sample. In analyses stratified by potential effect modifiers (Fig. 2 and 3), the negative associations between dietary quality scores and BP were consistent in most strata except for urbanicity. Specifically, the negative associations between diet scores and BP were greater in the urban group than in the rural group.

In the sensitivity analysis (see online supplementary material, Supplemental Figure 3s–6s), the negative associations between dietary quality and BP were generally robust. The associations remained similar when we changed our inclusion and exclusion criteria: (a) including participants who reported physician-diagnosed hypertension and the use of antihypertensive medication and (b) further excluding participants who reported being previously diagnosed with hypertension, diabetes, hyperlipidemia, CHD, stroke or cancer by a physician). In addition, the associations between dietary quality and BP results were unaffected when we ran a complete case analysis instead of performing multiple imputations. Finally, the general additive models developed using a spline also showed a linear relationship between BP and diet scores (treated as a continuous variable), which was consistent with the trend presented in Fig. 1.

## Discussion

In this large population study performed in a less-developed area in China, our results showed that higher adherence to DASH-style diet and aMED was associated with lower SBP and DBP. The antihypertensive effect of the DASH score was stronger than that of the aMED score. We also observed that the beneficial effect of healthy diet on SBP was greater in the hypertension subgroup than in either of the other BP subgroups.

Our results are generally consistent with previous findings showing that DASH and Mediterranean scores are negatively associated with BP. A longitudinal analysis in the SU.VI.MAX cohort in France found that DASH scores were significantly associated with a reduced SBP and DBP at baseline and a reduced BP increase after a 5-year follow-up^(30)^. The EPIC study, using data from Greek participants, found that the MED score had significant negative associations with both SBP and DBP^(31)^. Although our results suggest a stronger effect on BP than previous cross-sectional studies^(30,32,33)^, the beneficial effect is still modest from an individual perspective. However, a relatively small reduction in BP, if applied to an entire population, could have an enormous beneficial impact on public health^(34)^. In line with other studies^(12,35)^, our study also demonstrated that adherence to DASH-style diet and aMED is associated with a lower SBP within the usual range of BP, with a larger degree in the hypertension group than in the normotension or prehypertension group. The especially pronounced association between dietary quality and BP in the hypertension group may help facilitate the formulation of guidelines in less-developed regions to recommend dietary modifications, especially in individuals with hypertension.

In this study, we found that the DASH-style diet showed a larger protective effect on BP than aMED, as has been reported by an umbrella review and a meta-analysis^(12,13)^. Differences in associations between diet scores and BP may be attributable to differences in the food and nutrient components of each index. The DASH-style diet score awards points to diets high in dairy products and low in Na intake, whereas the aMED score awards points to diets with a high ratio of monounsaturated to saturated fat and high fish intake. Our investigations of the relative contribution of the individual components of diet indicated that total dairy products accounted for a large proportion of the beneficial effect of the DASH-style diet. Although findings from the original DASH diet have revealed the benefits of low-fat dairy foods combined with a diet high in fruits and vegetables on BP^(36)^, a meta-analysis showed that total dairy products were inversely associated with the risk of hypertension or elevated BP^(37)^. Considering that low-fat dairy accounts for a relatively small proportion of the total consumption of dairy products in most low- and middle-income countries such as China compared with high-income countries^(38)^, our results imply that the modified DASH diet, which includes total dairy products rather than low-fat dairy products only, may also produce strong protective effects on BP in less-developed regions in China. Furthermore, as one of the typical characteristics of the aMED, a high MUFA:SFA ratio failed to show significant beneficial effects on BP in the food group analysis. The main contributor to the MUFA:SFA ratio in Mediterranean countries is olive oil, which has high oleic acid and antioxidant polyphenol content^(22,39)^. However, owing to the inaccessibility of high-quality sources of MUFA (such as olive oil or marine fish) in Southwestern China, a high MUFA:SFA ratio in our sample could only reflect a high intake of vegetable oils, possibly accounting for the moderate effects of aMED on BP. In conclusion, although the intake of DASH components in our study was different from the intake of those foods in Western developed countries^(21)^, adherence to the DASH-style diet still demonstrated a significant beneficial effect on BP in our study. Our results imply that DASH-style diet is superior to aMED as dietary guidance for reducing BP in less-developed regions. Other studies have also reported that healthy diet patterns are more strongly associated with SBP than with DBP^(11,12)^. Recent studies showed a declining trend in the relative importance of DBP with age, with this variable being important predominantly in people under 45 years of age^(40)^, whereas a corresponding increase in the importance of systolic pressure with increasing age. In addition, the Framingham Study revealed that SBP is a better predictor of complications and death than DBP^(40)^. The mean age of our study population was 50·5 years, and more than half of the participants were elderly. Therefore, although there were some null associations between the diet scores and DBP in the BP subgroups, our findings indicated that adherence to DASH-style diet and aMED was associated with a significantly reduced SBP, which suggested that the adoption of healthy diet would be a useful measure for the control of BP in populations with suboptimal BP.

To our knowledge, this is the first large-scale epidemiological study to examine the association of adherence to DASH-style diet and aMED with specific BP values (SBP and DBP) in the general population in LEMR, whereas numerous studies have focused on populations with hypertension or elevated BP in developed countries. In addition, the selection of potential confounders was guided by the protocol of ESC-DAGs, which are under a framework of causal inference. Finally, the quality of the dietary data is guaranteed by the validated FFQ, stringent data audit and audio reviews.

Our study has several limitations. First, for feasibility, the FFQ used to assess food intake only consisted of thirteen food groups, which may not precisely capture the detailed dietary information of our participants. However, considering that many participants from LEMR speak different local languages and consume distinct foods (many foods are not included in any existing food database), some are even illiterate, a food group-based and simplified food questionnaire may be the only option that can ensure the efficiency of communication, the cooperation of participants and the comparability among various regions. Second, given the food group-based questionnaire and the cultural background and dietary habits of populations, we used the absolute weight in gram to measure the intakes of thirteen crude food groups rather than serving size, which may fail to consider the variation of weight of different foods for one serving equivalent. Third, we did not perform measurement error correction in this analysis, as the overwhelming majority of current statistical approaches were suitable only for single-food items or nutrients^(41)^. Forth, this study is a cross-sectional design using baseline data and does not allow the establishment of causal associations. However, we excluded participants with self-reported hypertension and use of antihypertensive medication to avoid the potential reverse causality that could be introduced by changes in habitual diet in response to the diagnosis of hypertension. In addition, we used a causal diagram and back door criteria to systematically control for confounders. Fifth, although we used an explicit way to explore the relative contribution of each component in these index, the estimation process would become very unstable (a subtle change in the effect could even operate in different directions) when the denominator (the overall effect) was close to null. This phenomenon is more apparent in the relative contribution of food component on aMED than DASH-style diet. So we recommended that the results should be interpreted cautiously when the overall association was weak. Last, all participants in this study were from China; therefore, it may not be possible to extrapolate our results to other LEMR. Future studies are needed to examine this association in other racial and national groups.

## Conclusions

In conclusion, our findings provide support for the recommendations of clinical guidelines on dietary strategy, showing that adherence to healthy diet such as the DASH-style diet and aMED is associated with lower BP in LEMR, especially among people with hypertension. Importantly, the DASH-style diet may be a more appropriate recommendation than aMED for controlling BP. This is true for patients who are in the hypertensive range as well as the prehypertensive and normotensive ranges. Prospective observational studies or clinical trials are needed to further investigate how the degree of adherence to the DASH-style diet and aMED can control BP in people in LEMR.

## Supplementary material

For supplementary material accompanying this paper visit https://doi.org/10.1017/S1368980022000106. click here to view supplementary material

## References

[1] Murray CJL , Aravkin AY , Zheng P et al. (2020) Global burden of 87 risk factors 204 countries territories, 1990–2019: a systematic analysis for the Global Burden of Disease Study 2019. Lancet 396, 122349. 10.1016/S0140-6736(20)30752-2 PMC7566194 33069327

[2] Lewington S , Clarke R , Qizilbash N et al. (2002) Age-specific relevance of usual blood pressure to vascular mortality: a meta-analysis of individual data for one million adults in 61 prospective studies. Lancet 360, 1903–1913. 12493255 10.1016/s0140-6736(02)11911-8

[3] Bennett JE , Stevens GA , Mathers CD et al. (2018) NCD Countdown 2030: worldwide trends in non-communicable disease mortality and progress towards Sustainable Development Goal target 3.4. Lancet 392, 1072–1088. 30264707 10.1016/S0140-6736(18)31992-5

[4] Collaboration NCDRF (2017) Worldwide trends in blood pressure from 1975 to 2015: a pooled analysis of 1479 population-based measurement studies with 19·1 million participants. Lancet 389, 37–55. 27863813 10.1016/S0140-6736(16)31919-5 PMC5220163

[5] Dong B , Arnold LW , Peng Y et al. (2016) Ethnic differences in cardiometabolic risk among adolescents across the waist–height ratio spectrum: National Health and Nutrition Examination Surveys (NHANES). Int J Cardiol 222, 622–628. 27517651 10.1016/j.ijcard.2016.07.169

[6] Di Cesare M , Khang Y-H , Asaria P et al. (2013) Inequalities in non-communicable diseases and effective responses. Lancet 381, 585–597. 23410608 10.1016/S0140-6736(12)61851-0

[7] Niessen LW , Mohan D , Akuoku JK et al. (2018) Tackling socioeconomic inequalities and non-communicable diseases in low-income and middle-income countries under the sustainable development agenda. Lancet 391, 2036–2046. 29627160 10.1016/S0140-6736(18)30482-3

[8] Attaei MW , Khatib R , McKee M et al. (2017) Availability and affordability of blood pressure-lowering medicines and the effect on blood pressure control in high-income, middle-income, and low-income countries: an analysis of the PURE study data. Lancet Public Health 2, e411–e419. 29253412 10.1016/S2468-2667(17)30141-X

[9] Appel LJ , Brands MW , Daniels SR et al. (2006) Dietary approaches to prevent and treat hypertension: a scientific statement from the American Heart Association. Hypertension 47, 296–308. 16434724 10.1161/01.HYP.0000202568.01167.B6

[10] Whelton PK , Carey RM , Aronow WS et al. (2018) 2017 ACC/AHA/AAPA/ABC/ACPM/AGS/APhA/ASH/ASPC/NMA/PCNA Guideline for the Prevention, Detection, Evaluation, and Management of High Blood Pressure in Adults: executive summary: a report of the American College of Cardiology/American Heart Association Task Force on Clinical Practice Guidelines. Circulation 138, e426–e483. 30354655 10.1161/CIR.0000000000000597

[11] Ndanuko RN , Tapsell LC , Charlton KE et al. (2016) Dietary patterns and blood pressure in adults: a systematic review and meta-analysis of randomized controlled trials. Adv Nutr 7, 76–89. 26773016 10.3945/an.115.009753 PMC4717885

[12] Gay HC , Rao SG , Vaccarino V et al. (2016) Effects of different dietary interventions on blood pressure: systematic review and meta-analysis of randomized controlled trials. Hypertension 67, 733–739. 26902492 10.1161/HYPERTENSIONAHA.115.06853

[13] Sukhato K , Akksilp K , Dellow A et al. (2020) Efficacy of different dietary patterns on lowering of blood pressure level: an umbrella review. Am J Clin Nutr 112, 1584–1598. 33022695 10.1093/ajcn/nqaa252

[14] Popkin BM (2006) Global nutrition dynamics: the world is shifting rapidly toward a diet linked with noncommunicable diseases. Am J Clin Nutr 84, 289–298. 16895874 10.1093/ajcn/84.1.289

[15] Tucker KL (2010) Dietary patterns, approaches, and multicultural perspective. Appl Physiol Nutr Med 35, 8. 10.1139/H10-010 20383235

[16] Zhao X , Hong F , Yin J et al. (2021) Cohort profile: the China Multi-Ethnic Cohort (CMEC) study. Int J Epidemiol 50, 721–721l. 33232485 10.1093/ije/dyaa185 PMC8271196

[17] National Institute for Nutrition and Health (2018) China Food Composition Tables. 6th ed. Beijing: Peking University Medical Press.

[18] National Health Commission of the People’s Republic of China (2013) Dietary Guide for Adult Diabetes Patients (WS/T 429–2013). Beijing: National Health Commission of the People’s Republic of China.

[19] Xiao X , Qin Z , Lv X et al. (2021) Dietary patterns and cardiometabolic risks in diverse less-developed ethnic minority regions: results from the China Multi-Ethnic Cohort (CMEC) Study. Lancet Reg Health West Pac 15, 100252. 34528018 10.1016/j.lanwpc.2021.100252 PMC8383007

[20] Chiu S , Bergeron N , Williams PT et al. (2016) Comparison of the DASH (Dietary Approaches to Stop Hypertension) diet and a higher-fat DASH diet on blood pressure and lipids and lipoproteins: a randomized controlled trial. Am J Clin Nutr 103, 341–347. 26718414 10.3945/ajcn.115.123281 PMC4733264

[21] Fung TT , Chiuve SE , McCullough ML et al. (2008) Adherence to a DASH-style diet and risk of coronary heart disease and stroke in women. Arch Intern Med 168, 713–720. 18413553 10.1001/archinte.168.7.713

[22] Trichopoulou A , Costacou T , Bamia C et al. (2003) Adherence to a Mediterranean diet and survival in a Greek population. N Engl J Med 348, 2599–2608. 12826634 10.1056/NEJMoa025039

[23] Shan ZL , Li YP , Baden MY et al. (2020) Association between healthy eating patterns and risk of cardiovascular disease. JAMA Intern Med 180, 1090–1100. 32539102 10.1001/jamainternmed.2020.2176 PMC7296454

[24] Fung TT , McCullough ML , Newby PK et al. (2005) Diet-quality scores and plasma concentrations of markers of inflammation and endothelial dysfunction. Am J Clin Nutr 82, 163–173. 16002815 10.1093/ajcn.82.1.163

[25] Chobanian AV , Bakris GL , Black HR et al. (2003) The seventh report of the joint national committee on prevention, detection, evaluation, and treatment of high blood pressure: the JNC 7 report. JAMA 289, 2560–2572. 12748199 10.1001/jama.289.19.2560

[26] Ferguson KD , McCann M , Katikireddi SV et al. (2020) Evidence Synthesis for Constructing Directed Acyclic Graphs (ESC-DAGs): a novel and systematic method for building directed acyclic graphs. Int J Epidemiol 49, 322–329. 31325312 10.1093/ije/dyz150 PMC7124493

[27] Pearl J , Glymour M & Jewell NP (2016) Causal Inference in Statistics: A Primer. West Sussex: Wiley.

[28] Trichopoulou A , Bamia C & Trichopoulos D (2009) Anatomy of health effects of Mediterranean diet: Greek EPIC prospective cohort study. BMJ – Br Med J 338, b2337. 19549997 10.1136/bmj.b2337 PMC3272659

[29] van Buuren S & Groothuis-Oudshoorn K (2011) Mice: multivariate imputation by chained equations in R. J Stat Softw 45, 1–67.

[30] Dauchet L , Kesse-Guyot E , Czernichow S et al. (2007) Dietary patterns and blood pressure change over 5-years follow-up in the SU.VI.MAX cohort. Am J Clin Nutr 85, 1650–1656. 17556705 10.1093/ajcn/85.6.1650

[31] Psaltopoulou T , Naska A , Orfanos P et al. (2004) Olive oil, the Mediterranean diet, and arterial blood pressure: the Greek European Prospective Investigation into Cancer and Nutrition (EPIC) study. Am J Clin Nutr 80, 1012–1018. 15447913 10.1093/ajcn/80.4.1012

[32] Lelong H , Blacher J , Menai M et al. (2016) Association between blood pressure and adherence to French Dietary Guidelines. Am J Hypertens 29, 948–958. 26908464 10.1093/ajh/hpw017

[33] Bendinelli B , Masala G , Bruno RM et al. (2019) A priori dietary patterns and blood pressure in the EPIC Florence cohort: a cross-sectional study. Eur J Nutr 58, 455–459. 29951936 10.1007/s00394-018-1758-2

[34] Krauss RM , Eckel RH , Howard B et al. (2000) AHA Dietary Guidelines: revision 2000: a statement for healthcare professionals from the Nutrition Committee of the American Heart Association. Circulation 102, 2284–2299. 11056107 10.1161/01.cir.102.18.2284

[35] Saneei P , Salehi-Abargouei A , Esmaillzadeh A et al. (2014) Influence of Dietary Approaches to Stop Hypertension (DASH) diet on blood pressure: a systematic review and meta-analysis on randomized controlled trials. Nutr Metab Cardiovasc Dis 24, 1253–1261. 25149893 10.1016/j.numecd.2014.06.008

[36] Svetkey LP , Simons-Morton D , Vollmer WM et al. (1999) Effects of dietary patterns on blood pressure: subgroup analysis of the Dietary Approaches to Stop Hypertension (DASH) randomized clinical trial. Arch Intern Med 159, 285–293. 9989541 10.1001/archinte.159.3.285

[37] Soedamah-Muthu SS , Verberne LD , Ding EL et al. (2012) Dairy consumption and incidence of hypertension: a dose-response meta-analysis of prospective cohort studies. Hypertension 60, 1131–1137. 22987924 10.1161/HYPERTENSIONAHA.112.195206

[38] Dehghan M , Mente A , Rangarajan S et al. (2018) Association of dairy intake with cardiovascular disease and mortality in 21 countries from five continents (PURE): a prospective cohort study. Lancet 392, 2288–2297. 30217460 10.1016/S0140-6736(18)31812-9

[39] Massaro M , Scoditti E , Carluccio MA et al. (2020) Effects of olive oil on blood pressure: epidemiological, clinical, and mechanistic evidence. Nutrients 12, 1548. 32466599 10.3390/nu12061548 PMC7352724

[40] Kannel WB , Gordon T & Schwartz MJ (1971) Systolic *v.* diastolic blood pressure and risk of coronary heart disease. The Framingham study. Am J Cardiol 27, 335–346. 5572576 10.1016/0002-9149(71)90428-0

[41] Bennett DA , Landry D , Little J et al. (2017) Systematic review of statistical approaches to quantify, or correct for, measurement error in a continuous exposure in nutritional epidemiology. BMC Med Res Methodol 17, 146. 28927376 10.1186/s12874-017-0421-6 PMC5606038

