# Supplementary material for: Association of Dietary Approaches to Stop Hypertension diet and Mediterranean diet with blood pressure in less-developed ethnic minority regions
Source: Public Health Nutr. 2022 Jan 14;25(12):3476–86. doi: 10.1017/S1368980022000106 (PMC9991676; doi:10.1017/S1368980022000106)
Supplement: Supplementary file 1 [file S1368980022000106sup.zip › S1368980022000106sup001.docx]

**Contents**

[**Text 1s.** The process of constructing directed acyclic graphs (DAG) 1](#_Toc91533669)

[**Text 2s.** The process of calculating the contribution of dietary components 1](#_Toc91533670)

[**Table 1s.** Scoring criteria for the DASH-style diet score and mean intake of related food group across quintiles 3](#_Toc91533671)

[**Table 2s.** Scoring criteria for the aMED score and mean intake of related food group across quintiles 4](#_Toc91533672)

[**Table 3s.** Blood pressure associated with 25% increment^*^ in DASH-style diet score and after alternate subtraction of each of its dietary component 5](#_Toc91533673)

[**Table 4s.** Blood pressure associated with 25% increment^*^ in aMED and after alternate subtraction of each of its dietary component 6](#_Toc91533674)

[**Figure 1s.** Participant flowchart. 7](#_Toc91533675)

[**Figure 2s.** The final constructed DAG. 8](#_Toc91533676)

[**Figure 3s.** Estimated associations between dietary quality and blood pressure by excluding the self-reported physician diagnosed diabetes, hyperlipidemia, coronary heart disease, stroke and cancer. (n=68341) 9](#_Toc91533677)

[**Figure 4s.** Estimated associations between dietary quality and blood pressure without excluding self-reported hypertension and use of anti-hypertensive medications. (n=91452) 10](#_Toc91533678)

[**Figure 5s.** Estimated associations between dietary quality and blood pressure without multiple imputation of the NA value of dietary intakes. (n=60212) 11](#_Toc91533679)

[**Figure 6s.** Estimated associations between dietary quality and blood pressure by using a smoothing spline. 12](#_Toc91533680)

## **Text 1s.** The process of constructing directed acyclic graphs (DAG)

The DAG was built based on the protocol of “Evidence Synthesis for Constructing Directed Acyclic Graphs” (ESC-DAGs), which combined evidence synthesis strategies and causal inference principles. [1] First, we determined a pool of potential confounders according to systematic literature review. Second, we assumed a saturated DAG by drawing directed or undirected edges between all variables, i.e., assuming that there was causal association between each pair of exposure, outcome and confounding factors. Third, each edge in the saturated DAG was assessed using several causal criteria (including temporality, validity, and theoretical support) and determined as retained, reversed, bi-directional or deleted. Fourth, a simplified DAG was constructed, thereby a series of conditional independences were generated according to the constructed DAG. Lastly, we continuously did the independence test and modified the DAG if the conditional independence did not agree with our data, until all the implied conditional independences were satisfied and the final DAG was reached. The final DAG can be found in Figure 1s.

According to the final DAG and back door criteria, the minimal sufficient set of confounders includes sex, age, marital status, highest education attained, household income, profession, regular smoking, physical activity, total energy intake, regular intake of sweeten beverage, insomnia symptoms, depressive symptom, anxiety symptom, menopause status for women, and family history of hypertension. In addition, we adjusted for regional level confounders (includes urbanicity and ethnicity that is also equivalent to the study sites or locations) and dieted-related variables that was not included in the analysis (i.e., regular intake of dietary supplements, regular intake of spicy food, regular intake of pepper food).

## **Text 2s.** The process of calculating the contribution of dietary components

To investigate the relative importance of the individual components of DASH-style diet and aMED in generating the associations between diet and blood pressure, we ran a food group analysis proposed by Trichopoulou[2,3]. Specifically, we assessed the contribution of each of the seven or eight components of DASH-style diet and aMED scores on blood pressure by dropping one component at a time from the total score, and then estimating the associations of the subtracted total scores (25% score range increment) with blood pressure by adjusting for the same confounders in the main analysis as well as the corresponding subtracted component. Then the relative importance of specific component can be calculated as reduction in apparent effect between the original total score and the subtracted total scores. Due to the score range would shorten after dropping one component, we multiplied the estimated coefficients of logistic regression by 25/29 for DASH-style diet and 29/33 for aMED to assure comparability.

**References**

1. Lewsey JD, Smith DJ, Green MJ, et al. Evidence synthesis for constructing directed acyclic graphs (ESC-DAGs): a novel and systematic method for building directed acyclic graphs[J]. *Int J Epidemiol* 2020, **49**(1):322-329. doi:10.1093/ije/dyz150.

2. Trichopoulou A, Bamia C, Trichopoulos D. Anatomy of health effects of Mediterranean diet: Greek EPIC prospective cohort study. *BMJ* 2009; **338**: b2337.

3. InterAct C, Romaguera D, Guevara M, et al. Mediterranean diet and type 2 diabetes risk in the European Prospective Investigation into Cancer and Nutrition (EPIC) study: the InterAct project. *Diabetes care* 2011; **34**(9): 1913-8.

## **Table 1s.** Scoring criteria for the DASH-style diet score and mean intake of related food group across quintiles

| **Component** | **Foods** | **Scoring criteria** | **Q1**  **g/day** | **Q5**  **g/day** |
| --- | --- | --- | --- | --- |
| Fruit | All fresh fruit | Q1=1 point  Q2=2 points  Q3=3 points  Q4=4 points  Q5=5 points | 6.49 | 360.93 |
| Vegetable | All fresh vegetables except tubers and legumes |  | 91.90 | 636.08 |
| Legumes | Soybeans, black beans, tofu, soybean milk, dried beans, dried bean curd |  | 0.00 | 39.19 |
| Dairy Product | Fresh milk, yogurt, cheese, milk tea |  | 0.0 | 1541.0 |
| Whole Grains | Oats, sorghum, dried corn, highland barely |  | 0.00 | 89.88 |
| Red & processed meat | Beef, mutton, pork and their products | Reverse score:  Q1=5 points  Q2=4 points  Q3=3 points  Q4=2 points  Q5=1 point | 249.50 | 9.57 |
| Sodium | Sodium in salt and preserved vegetables |  | 4870mg | 1075mg |

Abbreviation: DASH for Dietary Approaches to Stop Hypertension; Q for quintile.

## **Table 2s.** Scoring criteria for the aMED score and mean intake of related food group across quintiles

| **Component** | **Foods** | **Scoring criteria** | **Q1**  **g/day** | **Q5**  **g/day** |
| --- | --- | --- | --- | --- |
| Vegetables | All fresh vegetables except tubers and legumes | Q1=1 point  Q2=2 points  Q3=3 points  Q4=4 points  Q5=5 points | 91.90 | 636.08 |
| Legumes | Soybeans, black beans, tofu, soybean milk, dried beans, dried bean curd |  | 0.00 | 39.19 |
| Fruit | All fresh fruits |  | 6.49 | 360.93 |
| Whole grains | Oats, sorghum, dried corn, highland barely |  | 0.00 | 89.88 |
| Fish | Fish and all kinds of seafood products |  | 0.00 | 50.25 |
| MUFA: SFA | From all kinds of foods and fats |  | 1.19 | 2.17 |
| Red & processed meats | Beef, mutton, pork and their products | Reverse score:  Q1=5 points  Q2=4 points  Q3=3 points  Q4=2 points  Q5=1 point | 249.50 | 9.57 |
| Ethanol | All alcoholic beverages | moderate alcohol intake criteria ^b^ | - | - |

Abbreviation: aMED for alternative Mediterranean diet; Q for quintile; MUFA: SFA for the ratio of monounsaturated fatty acids: saturated fatty acids.

^a^. Due to the lack of fatty acids for food groups in the China food exchange list, we made an exchange value table according to the common consumed food items in each food group in Southwest China and the 2018 China food composition tables.

^b^. According to the encouragement of moderate alcohol intake, the alcohol consumptions were categorized into five groups: (10,30], (0,10] or (30,40], 0 or (40,45], (45,50], and >50 grams per day for men; (5,15], (0,5] or (15,25], 0 or (25,30], (30,35], and >35 grams per day for women, and then we assigned descending scores of 1-5 to corresponding individuals.

## **Table 3s.** Blood pressure associated with 25% increment^*^ in DASH-style diet score and after alternate subtraction of each of its dietary component

| Dietary variable | SBP | | | DBP | | |
| --- | --- | --- | --- | --- | --- | --- |
|  | BP coefficient  (95% CI) | Reduction in  apparent effect^†^ (%) | P value | BP coefficient  (95% CI) | Reduction in  apparent effect^†^ (%) | P value |
| DASH overall | -1.63(-1.83,-1.43) | 0.00% | <0.001 | -0.67(-0.80,-0.55) | 0.00% | <0.001 |
| DASH minus coarse grain | -1.23(-1.40,-1.05) | 24.64% | <0.001 | -0.53(-0.64,-0.41) | 21.43% | <0.001 |
| DASH minus fresh fruits | -1.25(-1.42,-1.07) | 23.31% | <0.001 | -0.54(-0.65,-0.42) | 20.23% | <0.001 |
| DASH minus fresh vegetables | -1.39(-1.56,-1.23) | 14.35% | <0.001 | -0.54(-0.65,-0.43) | 19.87% | <0.001 |
| DASH minus red and processed meats | -1.50(-1.68,-1.33) | 7.56% | <0.001 | -0.63(-0.74,-0.51) | 6.72% | <0.001 |
| DASH minus soybean products | -1.57(-1.73,-1.40) | 3.75% | <0.001 | -0.61(-0.72,-0.51) | 8.71% | <0.001 |
| DASH minus dairy products | -1.08(-1.26,-0.90) | 33.63% | <0.001 | -0.44(-0.56,-0.33) | 33.97% | <0.001 |
| DASH minus sodium | -1.35(-1.52,-1.18) | 17.13% | <0.001 | -0.59(-0.70,-0.48) | 12.68% | <0.001 |

Abbreviation: DASH: Dietary Approaches to Stop Hypertension; SBP: systolic blood pressure; DBP: diastolic blood pressure.

^*^. We assumed a linear relationship with beta representing the risk change per 25% score range increment.

^†^. Reduction in apparent effect (%) = (β_DASH overall_- β_DASH minus component_)/ (β_DASH overall_ - 0) *100%.

## **Table 4s.** Blood pressure associated with 25% increment^*^ in aMED and after alternate subtraction of each of its dietary component

| Dietary variable | SBP | | | DBP | | |
| --- | --- | --- | --- | --- | --- | --- |
|  | BP coefficient  (95% CI) | Reduction in  apparent effect^†^ (%) | P value | BP coefficient  (95% CI) | Reduction in  apparent effect^†^ (%) | P value |
| aMED overall | -0.91(-1.14,-0.68) | 0.00% | <0.001 | -0.30(-0.45,-0.15) | 0.00% | <0.001 |
| aMED minus coarse grain | -0.45(-0.68,-0.22) | 50.52% | <0.001 | -0.13(-0.28,0.01) | 55.98% | 0.075 |
| aMED minus fish | -0.81(-1.04,-0.59) | 10.73% | <0.001 | -0.25(-0.39,-0.10) | 18.25% | 0.001 |
| aMED minus fresh vegetables | -0.80(-1.02,-0.58) | 12.19% | <0.001 | -0.20(-0.35,-0.06) | 32.74% | 0.005 |
| aMED minus soybean products | -0.85(-1.08,-0.63) | 6.12% | <0.001 | -0.28(-0.43,-0.13) | 7.28% | <0.001 |
| aMED minus fresh fruits | -0.51(-0.74,-0.29) | 43.69% | <0.001 | -0.16(-0.30,-0.02) | 47.18% | 0.03 |
| aMED minus MSratio | -1.13(-1.35,-0.91) | -24.00% | <0.001 | -0.49(-0.63,-0.35) | -63.38% | <0.001 |
| aMED minus red and processed meats | -1.16(-1.38,-0.93) | -27.29% | <0.001 | -0.35(-0.49,-0.21) | -16.16% | <0.001 |
| aMED minus alcohol | -0.88(-1.08,-0.67) | 3.54% | <0.001 | -0.31(-0.45,-0.18) | -4.35% | <0.001 |

Abbreviation: aMED: alternative Mediterranean-style diet; SBP: systolic blood pressure; DBP: diastolic blood pressure; MSratio: monounsaturated fatty acid:saturated fatty acid ratio.

^*^. We assumed a linear relationship with beta representing the risk change per 25% score range increment.

^†^. Reduction in apparent effect (%) = (β_aMED overall_ - β_aMED minus component_)/ (β_aMED overall_ - 0) *100%.


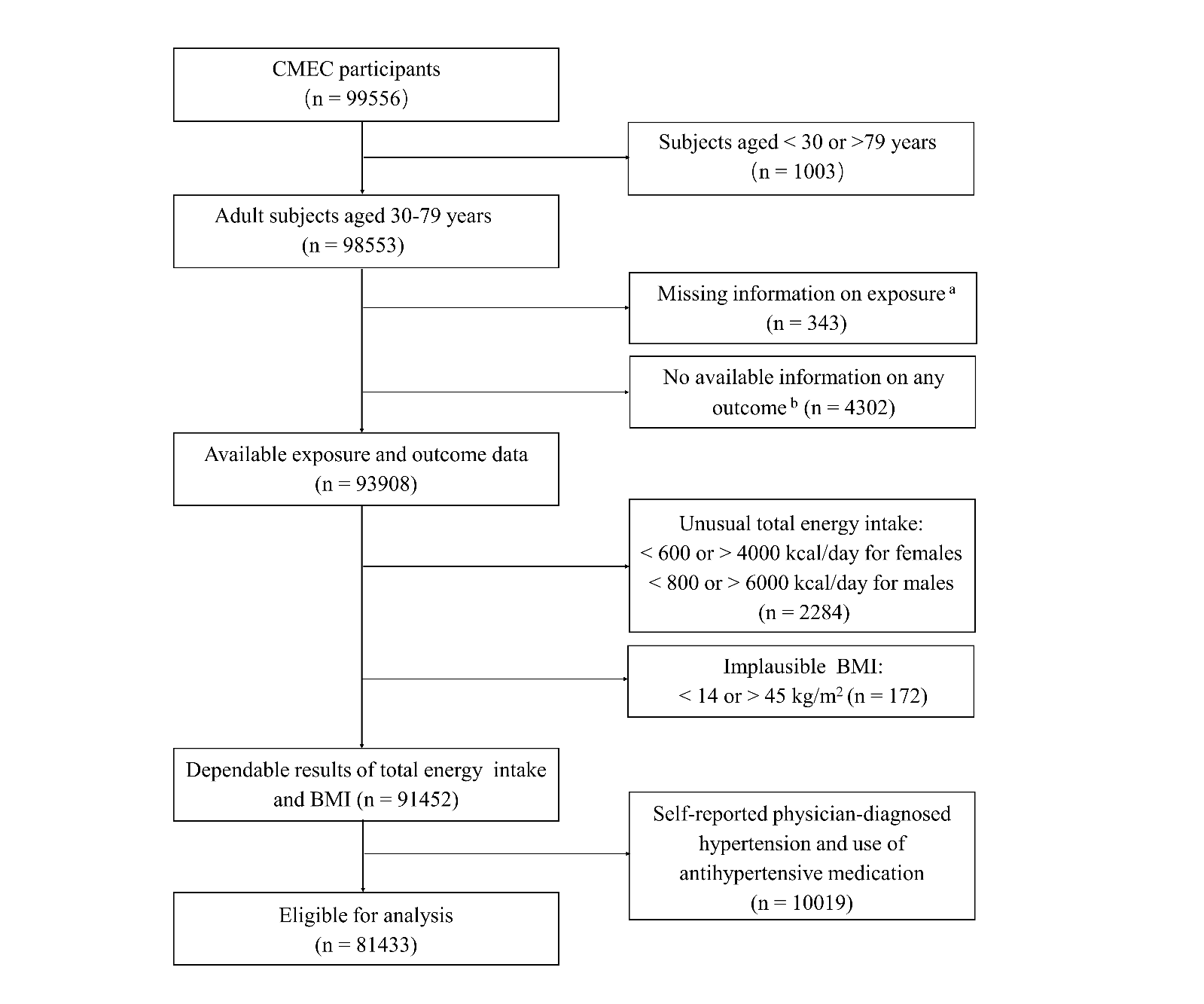


## **Figure 1s.** Participant flowchart.

CMEC, China Multi-Ethnic Cohort Study; BMI, body mass index. ^a^ Exposures included information about tea, alcohol and FFQ results; ^b^ Outcomes included physical measurements and blood tests.


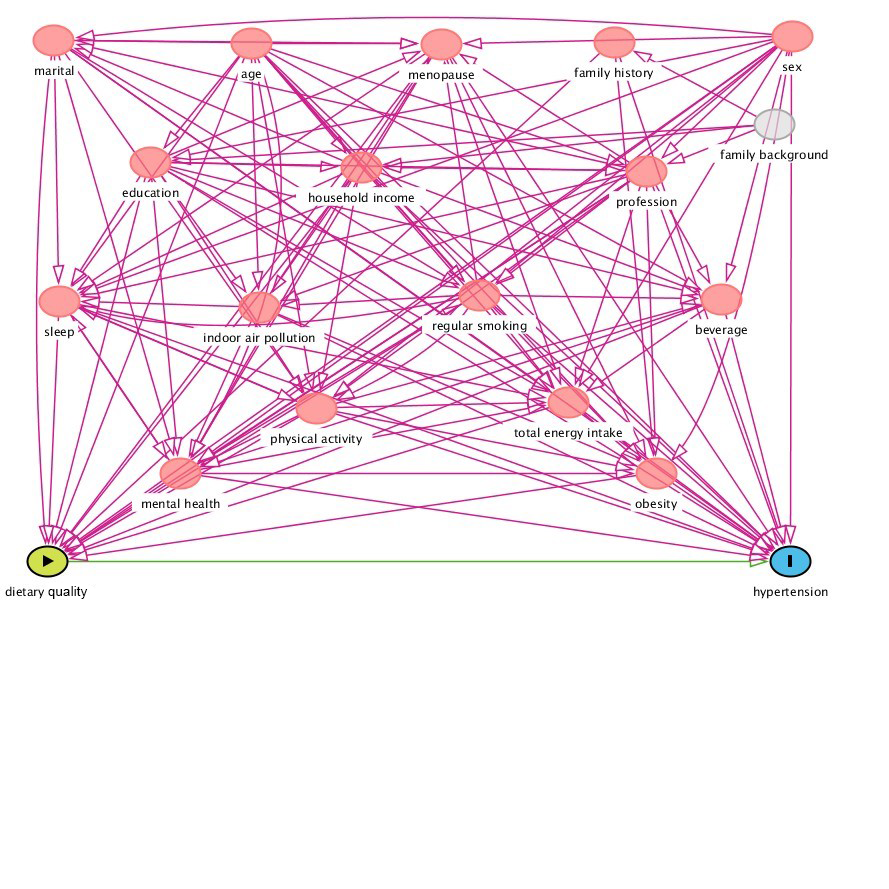


## **Figure 2s.** The final constructed DAG.

**
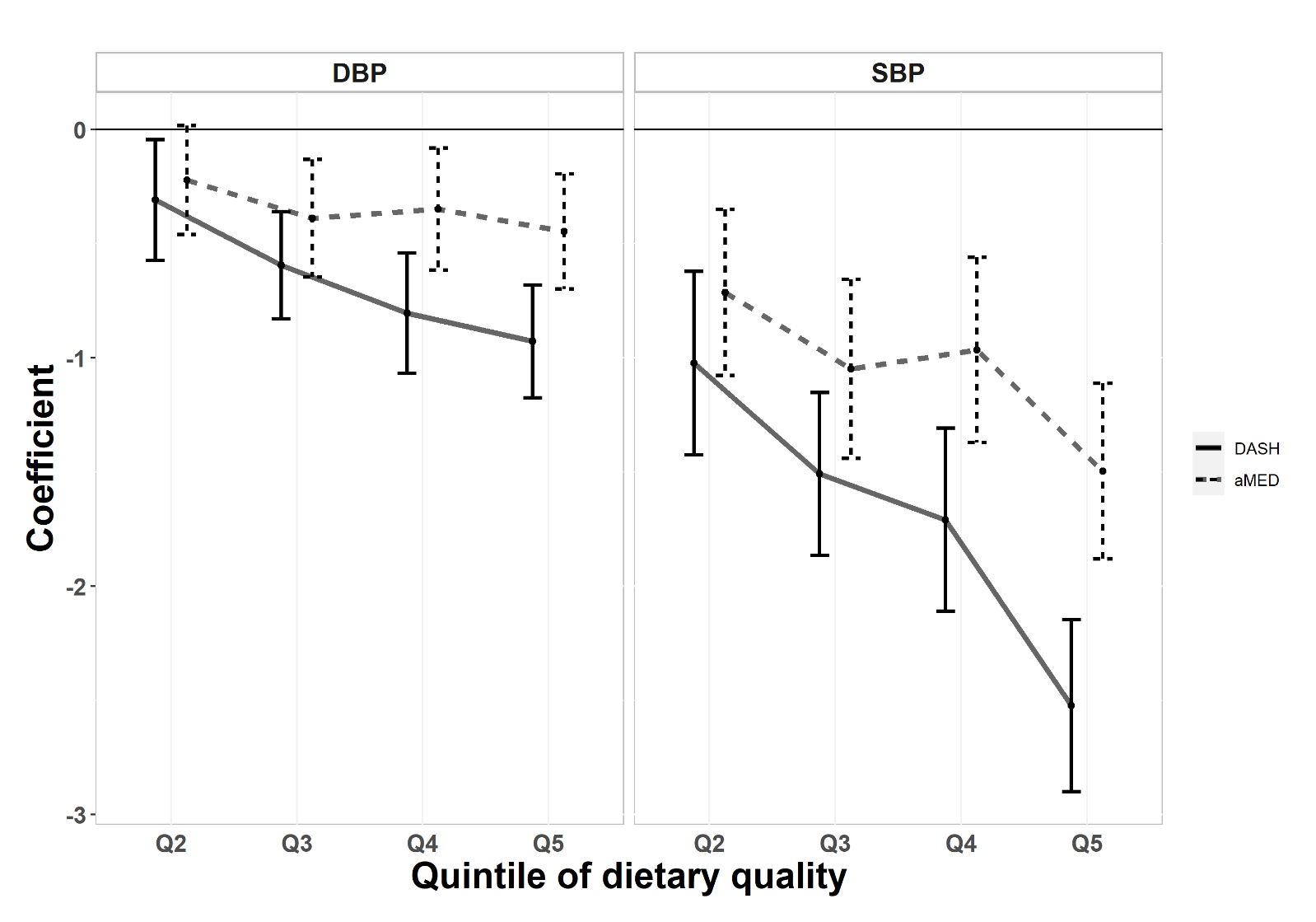
**

## **Figure 3s.** Estimated associations between dietary quality and blood pressure by excluding the self-reported physician diagnosed diabetes, hyperlipidemia, coronary heart disease, stroke and cancer. (n=68341)

**
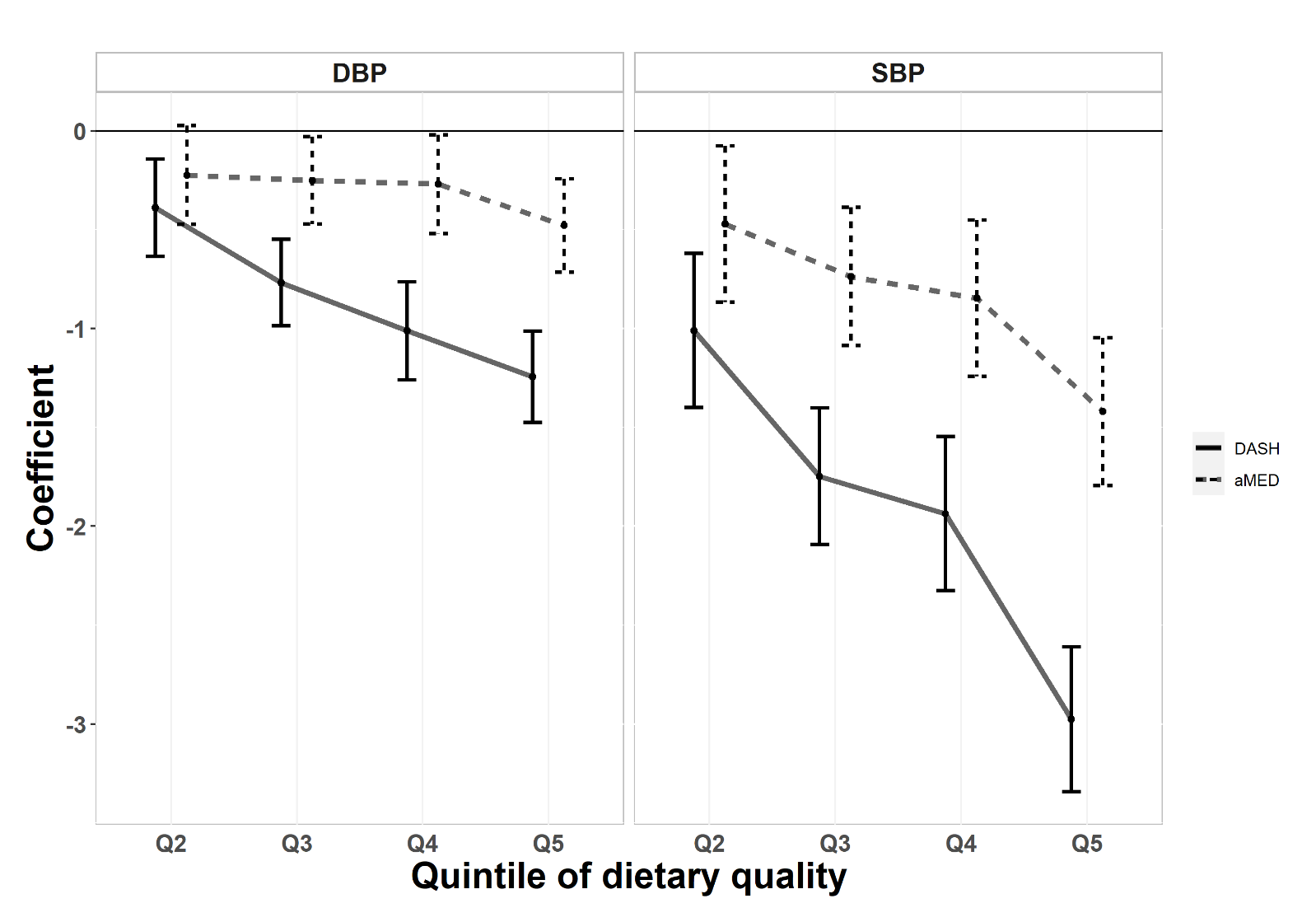
**

## **Figure 4s.** Estimated associations between dietary quality and blood pressure without excluding self-reported hypertension and use of anti-hypertensive medications. (n=91452)


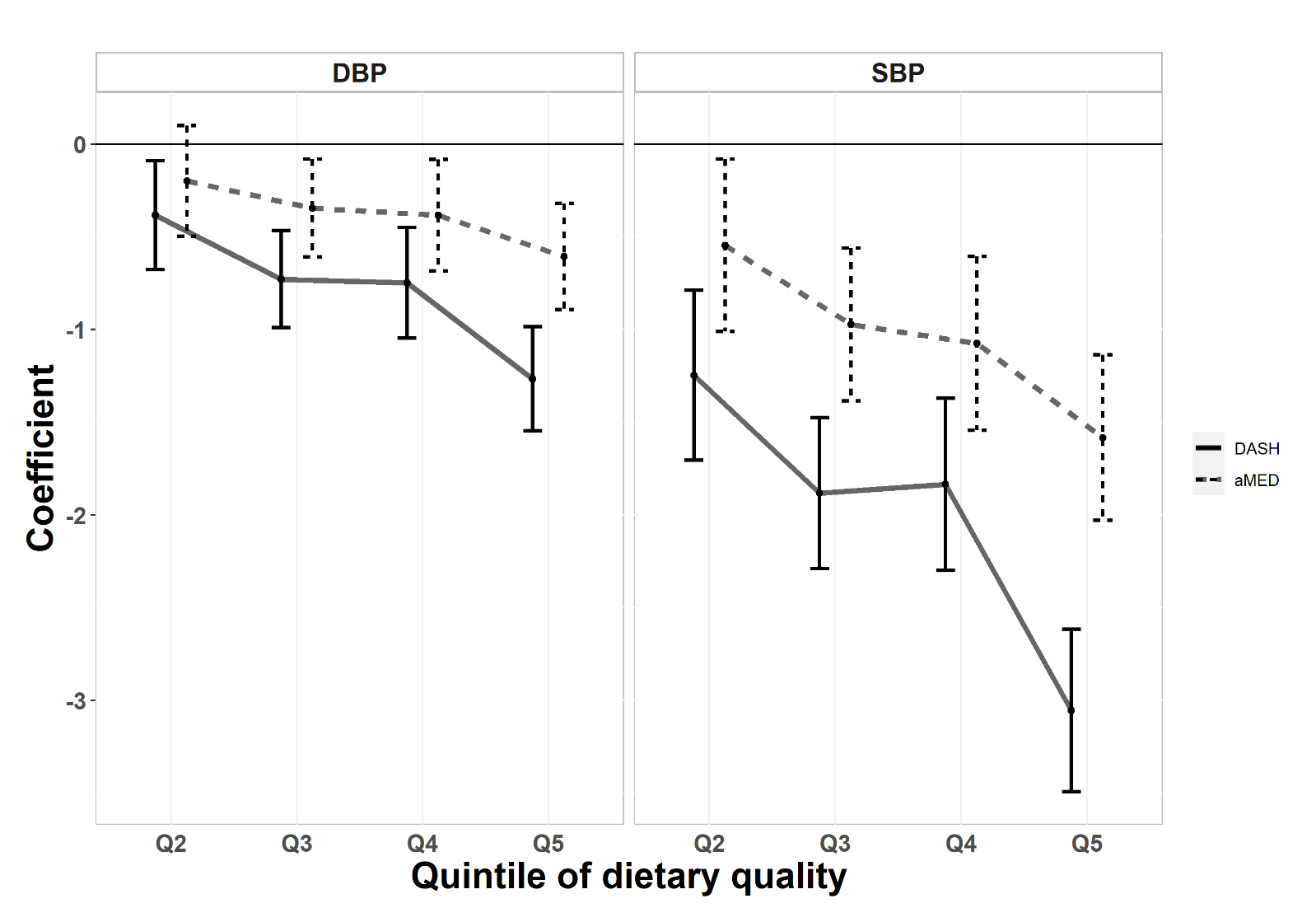


## **Figure 5s.** Estimated associations between dietary quality and blood pressure without multiple imputation of the NA value of dietary intakes. (n=60212)


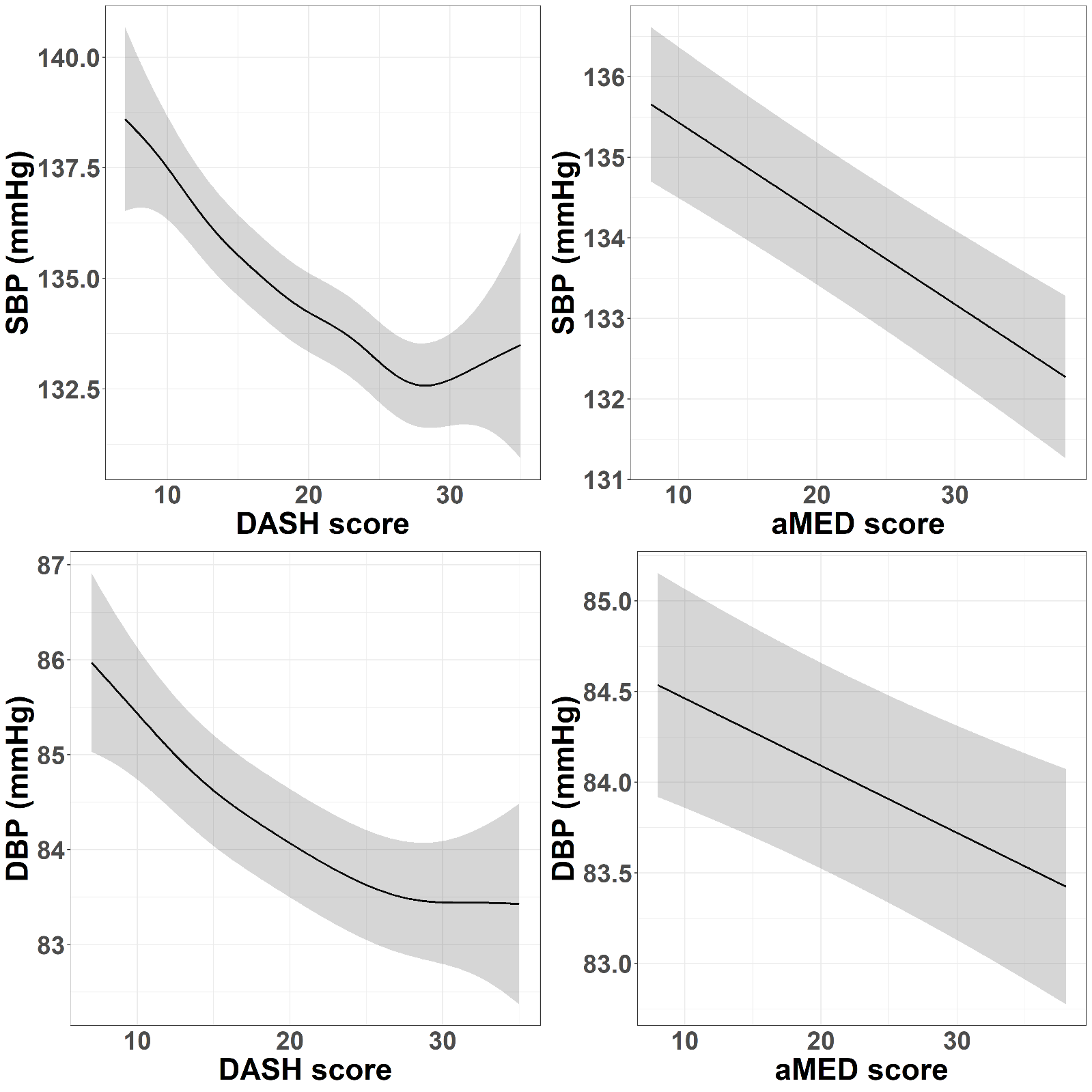


## **Figure 6s.** Estimated associations between dietary quality and blood pressure by using a smoothing spline.
